# Hypertension among Guatemalan women: the role of food insecurity and overweight/obesity

**DOI:** 10.3389/fnut.2026.1857055

**Published:** 2026-06-16

**Authors:** Paola A. Arevalo, María F. Kroker-Lobos, Félice Lê-Scherban, Manuel Ramirez-Zea, Karla Mesarina, Mireya Palmieri, Amy H. Auchincloss

**Affiliations:** 1 Urban Health Collaborative (UHC), Drexel University, Philadelphia, PA, United States; 2 Department of Epidemiology and Biostatistics, Dornsife School of Public Health, Drexel University, Philadelphia, PA, United States; 3 Research Center for the Prevention of Chronic Diseases (CIIPEC), Institute of Nutrition of Central America and Panama (INCAP), Guatemala City, Guatemala; 4 Secretariat of Food Security and Nutrition, Presidency of Guatemala, Guatemala City, Guatemala

**Keywords:** food insecurity, Guatemala, hypertension, Latin America, multinomial logistic regression, obesity, women

## Abstract

**Introduction:**

Food insecurity (FI) and obesity are associated with cardiovascular risk markers such as hypertension, yet it remains unclear whether their coexistence increases hypertension risk beyond their independent contributions. We hypothesized that women experiencing both FI and OWT/OB (‘coexisting burden’) would have higher odds of hypertension, compared to those experiencing only OWT/OB, only FI, or neither. We aimed to examine the association of FI and overweight/obesity (OWT/OB) with hypertension and systolic blood pressure (SBP) among Guatemalan women of reproductive age (15–49 years).

**Materials and methods:**

Data came from 1,538 women in Guatemala’s nationally representative 2018–2019 Health and Nutrition Epidemiological Surveillance System Survey (SIVESNU). A four-category exposure combined binary FI (assessed using the 8-item Food Insecurity Experience Scale) and OWT/OB status. SBP was measured and hypertension categorized using standard criteria. Multinomial logistic and linear regressions assessed associations with hypertension status and SBP, adjusting for covariates.

**Results:**

Participants had a mean age of 30 years; 76% experienced FI, 60% OWT/OB, 44% had both (‘coexisting burden’), and 32% had hypertension. Contrary to our hypothesis, associations were stronger among women with only OWT/OB (OR = 2.69, 95% CI: 1.44–5.02) than among those with coexisting burden (OR = 1.83, 95% CI: 1.01–3.31), compared to women with neither condition. No associations were found with pre-hypertension.

**Conclusion:**

Overall, OWT/OB, with or without FI, was associated with higher odds of hypertension, underscoring the need for community- and person-level interventions supporting healthier body weight.

## 1 Introduction

Within the United Nations’ Sustainable Developments Goal of Zero Hunger, specific targets aim to end hunger, achieve food security and end all forms of malnutrition by 2030 (1). Food insecurity (FI) is defined as “lacking physical and economic access to a sufficient and nutritious diet” (2, 3). As of 2023, an estimated 29% of the global population (2.33 billion) were experiencing moderate or severe FI, with a similar prevalence (28%) reported in Latin America and the Caribbean (1). Notably, FI has consistently been more prevalent among women than men, both globally and across all regions (1). At the same time, obesity rates have been rising at an alarming pace over the last decade, particularly in low- and middle-income countries where undernutrition and micronutrient deficiencies remain highly prevalent (1). Latin America and the Caribbean have the highest obesity prevalence, affecting nearly 30% of the adult population (1).

Obesity, a form of malnutrition, may result from excessive caloric intake due to inexpensive and energy-dense foods, as well as stress-induced metabolic changes (4–7). Its coexistence with FI has historically been described as paradoxical (the FI-obesity paradox), since FI was traditionally associated with undernutrition (8). While coping behaviors in response to FI—such as emotional eating (9) or irregular meal patterns (10, 11)—may influence body weight, current evidence suggests that, rather than reflecting a direct causal relationship, FI and obesity are more likely consequences of shared social disadvantages (7, 8). A complex interplay of social, economic, and psychological factors therefore underlies both FI and malnutrition (including obesity) (5, 8, 11–14). The short- and long-term health impacts of this interplay are not yet fully understood, highlighting the need for further investigation (7, 8, 14, 15). For example, while the separate influence of FI (16, 17) and obesity (18, 19) on cardiovascular risk markers such as hypertension are well-documented (20, 21), it is unclear whether their combined impact exacerbates hypertension risk beyond their independent contributions, particularly when measured clinically (16).

Despite growing evidence that FI and obesity go hand in hand, research on this coexisting burden and its implications for population health remains scarce in Latin American countries (19, 22). In Guatemala, a low-middle-income country in Central America, half of the population experiences food insecurity, malnutrition and poverty (23, 24). These conditions contribute to the growing burden of noncommunicable diseases (NCDs), the leading cause of death in the country (24). Recent reports have shown that nearly one-third of the population are affected by hypertension (24). Moreover, undernutrition (25), obesity (26), anemia (27) and hypertension (28) are highly prevalent among Guatemalan women of reproductive age (15–49 years old) (WRA), with reported socioeconomic and ethnic disparities in all forms of malnutrition (25–27). Nutritional inequalities among WRA increase their risk of impaired fertility, maternal health complications, chronic diseases, and ultimately higher mortality (29, 30). One study suggested that WRA with both FI and obesity faced an increased risk of excessive weight gain during pregnancy and postpartum (22). However, the impact of the coexisting burden of FI and obesity on other health outcomes among WRA in Latin America remains unexplored. Understanding the implications of this coexisting burden on cardiovascular disease risk among vulnerable populations like WRA has the potential to inform targeted interventions.

In this study, we aimed to estimate the joint association of FI and overweight/obesity (OWT/OB) with hypertension among Guatemalan WRA (15–49 years old) using nationally representative data. We hypothesized that women experiencing both FI and OWT/OB (‘coexisting burden’) would have higher odds of hypertension, compared to those experiencing only OWT/OB, only FI, or neither.

## 2 Materials and methods

### 2.1 Study design and population

This cross-sectional study aimed to estimate the joint association of FI and overweight/obesity (OWT/OB) with hypertension among Guatemalan WRA. We utilized data from WRA who participated in Guatemala’s 2018–2019 Health and Nutrition Epidemiological Surveillance System Survey (SIVESNU). SIVESNU is a semi-annual nationally representative survey designed to monitor health and nutrition in Guatemala (31). As part of the original SIVESNU sampling framework—designed to support surveillance objectives across women and young children—households were eligible for recruitment if they had at least one WRA (15–49 years) and one child under 5 years of age. SIVESNU adheres to a multistage sampling design to ensure national representativeness of eligible households. Details about the survey design are provided elsewhere (31). The 2018–2019 cycle is the most recent publicly available dataset as of writing.

#### 2.1.1 Analytic sample

For the present study, our analytic sample was restricted to non-pregnant women with complete information on exposure, outcome, and covariates. Out of 1755 WRA who participated in the 2018–2019 SIVESNU cycle, we excluded pregnant women (*N* = 95) and those with missing pregnancy status (*n* = 4), leaving 1,656 women eligible for analysis. We further excluded 118 participants with missing data on key variables (exposure, outcome and/or covariates), leaving 1,538 women in the analytic sample (93% of the eligible sample, see Supplementary Figure 1). Compared to those excluded, women included in the analysis were more physically active, had higher socioeconomic position (SEP), and had fewer children at home. However, included and excluded participants were similar in terms of hypertension status, urbanicity, and education level (see Supplementary Table 1).

### 2.2 Exposure: FI and body mass index

FI was assessed using the Food Insecurity Experience Scale (FIES) (32), a shortened scale largely based on the Latin American and Caribbean Food Security Scale (ELCSA) (33, 34). The FIES is an 8-item experience-based tool, with cross-cultural comparability across countries and population subgroups (35), and was validated by the Food and Agricultural Organization of the United Nations (32, 36). In SIVESNU, the FIES was administered at the household level using a 12-month recall period, with responses provided by the head of household. Response options for each item were ‘no’ (0) or ‘yes’ (1). We summed the 8 items and then classified the scale according to Cafiero et al. (36) as: food secure (score = 0), mild FI (score = 1–3), moderate FI (score = 4–6), severe FI (score = 7–8). See Supplementary Table 2 for the FIES questions.

Trained professionals measured each woman’s standing height using a portable stadiometer (ShorrBoard, Olney, Maryland) and weight using a digital floor scale (Seca 874 Digital Floor Scale, Olney, Maryland). Body mass index (weight in kg/height in meters squared, BMI) was categorized as less than overweight (<25.0 kg/m^2^), overweight (OWT, 25.0- < 30 kg/m^2^) or obese (OB ≥ 30.0 kg/m^2^).

We then constructed a variable that cross-classified FI and BMI categories to evaluate whether the combined exposure was associated with greater hypertension risk beyond the independent contribution of each condition. To obtain adequate sample sizes across cross-classified exposure and outcome categories, we dichotomized FI and BMI status. We defined FI as having at least mild FI (score > = 1) and high BMI status as being overweight or obese (BMI > =25.0 kg/m2). The final 4-category exposure variable was (1) not FI and not OWT/OB, (2) only FI, (3) only OWT/OB, (4) FI and OWT/OB (‘coexisting burden’).

### 2.3 Outcome: blood pressure

Participants provided information on hypertension diagnosis and use of antihypertensive medications. Trained clinicians measured blood pressure (BP) using MDF Lenus Digital Blood Pressure Monitor (MDF Instruments, Los Angeles, California) with an arm cuff placed on the left arm after the participant sat for at least 5 min. Three BP measurements were taken at one-min intervals, and the second and third measurements were averaged (31). BP was classified according to the 2017 American College of Cardiology/ American Heart Association (ACC/AHA) guidelines (37). Pre-hypertension was defined as SBP of 120–129 mm Hg or diastolic BP < 80 mm Hg. Hypertension was defined as SBP ≥ 130 mm Hg, diastolic BP ≥ 80 mm Hg, or currently taking antihypertensive medications. If blood pressure was unmeasured but the response was affirmative to antihypertensive medications, then we classified it as hypertension; otherwise, it was considered missing and excluded from the analysis.

### 2.4 Confounders

Confounders were selected *a priori* based on literature review and directed acyclic graphs (DAGs). Age, lactating status, and physical activity were included as confounders because they are known to be related to FI and obesity status (9, 11, 12, 22, 38), as well as with hypertension risk (16, 18, 19, 39–41), due to their influence on nutritional demands, metabolic changes, and chronic stress. These variables were not considered mediators in the causal pathway of interest.

We categorized age into four groups: 15–19 years, 20–29 years, 30–39 years, 40–49 years. Lactating status was included as a binary variable (lactating vs. not lactating). Physical activity was assessed using the Global Physical Activity Questionnaire (GPAQ) (42). Participants were asked separately about the frequency and duration of moderate and vigorous physical activity (MVPA) across work, sports, transportation (walking/cycling) and recreational domains. We calculated total MVPA (minutes per week) using standard GPAQ methods: (43) (MVPA = moderate activity minutes per week + [2 × vigorous intensity minutes per week]). We then categorized MVPA into tertiles (low, middle, and high). For linear regression models with SBP as the outcome, we additionally included ‘use of anti-hypertensive medication’ as a confounder as it directly suppresses blood pressure and could influence body weight.

Household-level confounders included number of adults, number of children, urbanicity, and socioeconomic position (SEP), because they are known to influence FI and dietary patterns (thus nutritional status/obesity) (9, 11, 12, 22, 38) and are also positively associated with hypertension through pathways related to stress (16, 18, 19, 39–41). We included separate variables for household number of adults and children, as they may contribute differently to household resource allocation and dietary patterns. We also included a binary measure of urbanicity determined based on the Guatemala’s National Institute of Statistics definition of urban areas as “cities, towns, villages, or populated places that have the category of a neighborhood or condominium, and those with more than 2,000 inhabitants, provided that 51% or more of the households have electric lighting and piped water (tap) within their residences (homes)” (44). Since household income information was not collected in SIVESNU, we derived an SEP index using 12 variables related to socioeconomic status including assets ownership (television, refrigerator, washing machine, landline phone, computer, microwave, vehicle), healthcare access (insurance, ever seen a dentist), home status (ownership vs. rental), number of children living in the household, and home environment (separate sleeping and cooking spaces) (28, 45). See Supplementary Table 3 for variable description and loadings. We applied principal components analysis (PCA), identifying a single factor that retained 9 binary variables and explained 47% of total variance. We then estimated the SEP index score by summing the 9 retained variables (unweighted), and categorized the score into tertiles (lower SEP, score 0–1; middle SEP, score 2–3; higher SEP, score 4–9).

### 2.5 Statistical methods

Descriptive analyses (unweighted frequencies and percentages) contrasted participant characteristics across the exposure and the outcome. Multinomial logistic regression was used to examine the relative odds of having pre-hypertension or hypertension (compared to normotension). Linear regression was used to model systolic blood pressure (SBP).

We fit a sequence of progressively adjusted models for each outcome. Model 1 was unadjusted. Model 2 was adjusted for individual-level confounders (age, physical activity, lactating status). Model 3 added SEP, number of children and adults in the household, and urban/rural residence. For linear regression models with continuous SBP as the outcome, all models were additionally adjusted for use of anti-hypertensive medication (*N* = 59, 3.8%) because it directly lowers measured blood pressure. Anti-hypertensive medication use was not included in multinomial logistic regression models, as medication use was part of the hypertension definition and adjustment could have resulted in over-adjustment.

All regression analyses incorporated SIVESNU’s complex survey design using variables that accounted for the primary sampling unit (conglomerado) and the sampling weight (pesomujer). A two-sided *p*-value <0.05 was considered statistically significant. All analysis were performed using SAS 9.4® (Statistical Analysis System, RRID: SCR_008567).

#### 2.5.1 Sensitivity analyses

We conducted two sensitivity analyses. First, we repeated the multinomial regression models using different hypertension cut-offs (46, 47) to test whether our conclusions hold across other hypertension guidelines (Supplementary Table 4). Second, we refined the cross-classification exposure by decomposing FI into mild, moderate, and severe, resulting in an 8-category exposure variable (Supplementary Table 5).

## 3 Results

### 3.1 Descriptive results

The mean age of the sample was 30.2 years (SD 9.3) (Table 1). Nearly 40% of the sample had high BP (7% pre-hypertension, 32% hypertension). Mean systolic blood pressure was 114.1 mm Hg (SD 13.6). Overall, 74% of women in the sample experienced FI and 60% had OWT/OB. Forty-four percent experienced coexisting burden of FI and OWT/OB, whereas 16% had only OWT/OB and 30% only FI. Women with coexisting burden were more likely to be older, live in a rural area, and middle SEP. Those experiencing only OWT/OB were disproportionately urban and of higher SEP, whereas those with only FI were disproportionately younger, resided in rural areas, and had lower SEP. Participants with high BP (pre-hypertensive and hypertensive) were older and predominantly lived in rural areas compared to normotensive individuals (Supplementary Table 6).

**Table 1:** Characteristics of the sample, stratified by food insecurity and overweight/obese status (*N* = 1,538)^a^.

		Food insecurity (FI) and overweight/obese (OWT/OB)			
	Total (*N* = 1,538)	Neither (*N* = 144, 9%)	Only FI (*N* = 467, 30%)	Only OWT/OB (*N* = 253, 16%)	Coexisting burden (FI and OWT/OB) (*n* = 674, 44%)
Hypertension					
Normal	946 (61%)	107 (74%)	358 (70%)	132 (52%)	349 (52%)
Pre-hypertension	100 (7%)	7 (1%)	28 (1%)	19 (1%)	46 (1%)
Hypertension	492 (32%)	30 (25%)	81 (29%)	102 (47%)	279 (47%)
SBP (mean ± SD)	114.1 ± 13.6	108.5 ± 11.6	110.2 ± 13.3	115.5 ± 11.6	117.4 ± 13.9
Food insecurity					
None	397 (26%)	144 (100%)	-	253 (100%)	-
Mild	612 (40%)	-	244 (52%)	-	368 (55%)
Moderate	364 (23%)	-	145 (31%)	-	219 (32%)
Severe	165 (11%)	-	78 (17%)	-	87 (13%)
BMI (mean ± SD)	27.0 ± 5.5	22.0 ± 2.1	22.0 ± 2.0	30.0 ± 4.0	30.4 ± 4.6
Age (mean ± SD)	30.2 ± 9.3	26.2 ± 8.6	26.1 ± 9.0	31.1 ± 8.6	33.6 ± 8.5
Age group					
15–19 years	240 (16%)	37 (26%)	139 (30%)	26 (10%)	38 (6%)
20–29 years	511 (33%)	65 (45%)	176 (38%)	88 (35%)	182 (27%)
30–39 years	480 (31%)	27 (19%)	97 (21%)	88 (35%)	268 (40%)
40–49 years	307 (20%)	15 (10%)	55 (12%)	51 (20%)	186 (28%)
Physical activity (tertiles)^b^					
Low	562 (37%)	72 (50%)	165 (35%)	87 (34%)	238 (36%)
Middle	497 (32%)	32 (22%)	161 (34%)	86 (34%)	218 (32%)
High	479 (31%)	40 (28%)	141 (31%)	80 (32%)	218 (32%)
Lactating status					
No	1,292 (84%)	116 (81%)	371 (79%)	217 (86%)	588 (87%)
Yes	246 (16%)	28 (19%)	96 (21%)	36 (14%)	86 (13%)
Socioeconomic position (tertiles)^c^					
Lower	533 (35%)	42 (29%)	216 (46%)	39 (15%)	236 (35%)
Middle	526 (34%)	41 (28%)	158 (34%)	72 (28%)	255 (38%)
Higher	479 (31%)	61 (43%)	93 (20%)	142 (57%)	183 (27%)
*N* children in household					
0 children	171 (11%)	19 (13%)	40 (9%)	30 (12%)	82 (12%)
1–2 children	790 (51%)	86 (60%)	230 (49%)	151 (60%)	323 (48%)
≥3 children	577 (38%)	39 (27%)	197 (42%)	72 (28%)	269 (40%)
*N* adults in household					
1–2 adults	841 (55%)	75 (52%)	254 (54%)	154 (61%)	358 (53%)
3–4 adults	479 (31%)	50 (35%)	245 (31%)	69 (27%)	215 (32%)
≥5 adults	218 (14%)	19 (13%)	68 (15%)	30 (12%)	101 (15%)
Urban/rural					
Urban	583 (38%)	64 (44%)	137 (29%)	133 (53%)	249 (27%)
Rural	955 (62%)	80 (56%)	330 (71%)	120 (47%)	425 (63%)

FI, food insecurity; OWT/OB, overweight/obese; SBP, systolic blood pressure; SD, standard deviation. ^a^Results presented in *N* (%), except for SBP and age presented as mean ± SD. ^b^Physical activity includes minutes/week of moderate and vigorous (multiplied by 2 to standardize to moderate) sports activity, as well as bicycle activity. Total minutes were summed and divided in tertiles as low (0–60), middle (61–210) and high (211–4,200). ^c^SEP was derived from nine variables that proxied household and individual assets/socio-economic position. See Supplementary Table 3.

### 3.2 Primary adjusted analysis

Table 2 displays adjusted regression analysis results for three-category hypertension and continuous systolic blood pressure. Statistically significant (*p* < 0.05) positive associations with hypertension were observed among those with ‘only OWT/OB’ and ‘coexisting burden,’ compared to women with neither FI nor OWT/OB. Although estimates were slightly attenuated after adjusting for confounders (Model 2 and Model 3), the associations remained consistent and statistically significant on both groups.

**Table 2:** Adjusted association between food insecurity and OWT/OB status, and outcomes for hypertension and systolic blood pressure (*N* = 1,538)^a^.

	Pre-hypertensive vs. normotensive^b^		Hypertensive vs. normotensive		SBP (mm Hg, continuous)	
	OR (95% CI)	P-value	OR (95% CI)	*p*-value	*β* Coeff. (95% CI)	*p*-value
Neither	Referent		Referent		Referent	
Model 1: Unadjusted						
Only FI	1.25 (0.36, 4.26)	0.72	0.86 (0.51, 1.45)	0.57	0.94 (−1.70, 3.57),	0.48
Only OWT/OB	2.89 (0.81, 10.39)	0.10	3.23 (1.76, 5.95)	<0.001	5.94 (2.94, 8.94)	<0.001
Coexisting burden	2.02 (0.65, 6.24)	0.22	2.89 (1.66, 5.03)	<0.001	7.16 (4.45, 9.87)	<0.001
Model 2: Adjusted for individual-level covariates^c^						
Only FI	1.31 (0.36, 4.72)	0.68	0.86 (0.49, 1.52)	0.60	0.94 (−1.77, 3.65)	0.49
Only OWT/OB	2.66 (0.73, 9.67)	0.14	2.54 (1.37, 4.70)	0.003	4.15 (1.22, 7.07)	0.01
Coexisting burden	1.70 (0.56, 5.14)	0.35	1.92 (1.07, 3.42)	0.03	4.59 (1.98, 7.18)	0.001
Model 3: Adjusted for Model 2 covariates + household-level covariates^d^						
Only FI	1.21 (0.32, 4.67)	0.78	0.79 (0.44, 1.43)	0.44	0.29 (−2.45, 3.02)	0.84
Only OWT/OB	2.79 (0.76, 10.32)	0.12	2.69 (1.44, 5.02)	0.002	4.63 (1.79, 7.51)	0.002
Coexisting burden	1.62 (0.50, 5.20)	0.42	1.83 (1.01, 3.31)	0.05	4.12 (1.47, 6.77)	0.003

CI, confidence interval; FI, food insecurity; OR, odds ratio; OWT/OB, overweight/obesity. ^a^All regression analysis accounted for survey weights. A two-sided *p*-value ≤0.05 is considered statistically significant. ^b^Outcome 1 (hypertension) was modeled with multinomial logistic regression. Outcome 2 (SBP) was modeled using linear regression. Hypertension status was defined according to AHA/ACC 2017 guidelines (pre-hypertension 120–129 and DBP <80, hypertension SBP≥130 or DBP≥80). ^c^Individual-level covariates: age group (15–19y, 20–29y, 30–39y, 40–49y), physical activity (tertiles of min/week), lactating status (yes/no). When modeling outcome 2 (SBP), the adjustment list also included hypertension medication (takes oral prescription medicine to control hypertension). ^d^Household-level covariates: socio-economic position (tertiles), number of children in the household (0, 1–2, ≥3), number of adults in the household (1–2, 3–4, ≥5), urbanicity (urban vs rural).

For example, compared to women who were normotensive and had neither FI nor OWT/OB, the odds of hypertension were 169% higher (model 3 OR = 2.69, 95%CI 1.44–5.02) among those with only OWT/OB, and 83% higher for those with coexisting burden (model 3 OR = 1.83, 95%CI 1.01–3.31). There was no evidence of a conditional association between only FI and hypertension (*p* > 0.4 for all models), nor between any category of the exposure and pre-hypertension (*p* > 0.1 for all models).

Results for systolic blood pressure (SBP) aligned with the hypertension results. Compared to the reference group, SBP was about 5 mmHg higher among those with only OWT/OB and 4 mmHg higher among those with the coexisting burden in fully adjusted models (model 3 Beta = 4.63, 95%CI 1.79–7.51; Beta = 4.12, 95%CI 1.47–6.77, respectively). As with hypertension, no association was observed between only FI and SBP (*p* > 0.4 for all models).

### 3.3 Sensitivity analysis

When repeating the multinomial regression models using different hypertension cut-offs (46, 47), we obtained similar estimates and same inference, suggesting that our results are not sensitive to the specific definition of hypertension used (Supplementary Table 4). When decomposing FI into mild, moderate, and severe categories within the OWT/OB cross-classification, only OWT/OB and mild coexisting burden remained significantly associated with hypertension and SBP. Moderate coexisting burden was significantly associated with SBP only. Although severe coexisting burden showed a similar estimate magnitude to the mild coexisting burden, the association was not statistically significant, likely due to limited sample size. Additionally, severe FI was associated with hypertension in the opposite direction (i.e., lower odds of hypertension), which could be attributable to small sample size and lower BMI in this subgroup (Supplementary Table 5).

## 4 Discussion

Overall, among a nationally representative sample of Guatemalan WRA, those who experienced coexisting burden of FI and OWT/OB, or who experienced only OWT/OB, had higher odds of hypertension and higher SBP compared to those with neither FI nor OWT/OB. Our descriptive findings highlight the high burden of chronic disease risk among WRA in Guatemala, and align with prior research reporting socioeconomic conditions and disparities in malnutrition in the country (25, 26). The majority of participants experienced FI (74%), and similarly the majority had OWT/OB (60%); nearly 40% had high blood pressure. The majority of the sample lived in rural areas (62%). Those experiencing the ‘coexisting burden’ of FI and OWT/OB were predominantly of middle SEP and lived in rural areas, as compared to those with only OWT/OB (higher SEP, urban) or only FI (lower SEP, rural).

Our results point to OWT/OB as the key driver of high BP among WRA in Guatemala, with FI as a contributor to high BP in combination with OWT/OB, but not on its own among under- or normal-weight WRA. We conjecture that women experiencing FI without OWT/OB might have a healthier, less-processed diet which may lower risk of hypertension (48). In Guatemala, FI may also reflect reduced overall caloric intake or limited access to processed, sodium-dense foods among some rural and lower-SEP households, which could partially attenuate hypertension risk despite economic hardship (49–51). The lack of an association between FI and hypertension is supported by a systematic review (59), which found no association between FI and clinically measured BP; however, there were no studies that assessed the association according to categories of BMI. Contrary to our hypothesis, we found stronger associations with hypertension and SBP among women with only OWT/OB than among those with the coexisting burden. Given that FI is typically more variable over time than nutritional status (5, 8, 52), it is possible that women with only OWT/OB had more consistent exposure to unhealthy dietary patterns, and other lifestyle factors that could have contributed to hypertension. Of note, a larger proportion of higher SEP was observed among those only OWT/OB (57%) compared to those with coexisting burden (27%), only FI (20%), or neither (47%). In addition, the majority of WRA with only OWT/OB lived in urban areas; this was not the case for the other exposure categories.

Although hypertension is highly prevalent in Latin American countries (53), evidence on its relationship with the food insecurity and obesity paradox remains scarce (7, 53). To our knowledge, this is the first study to examine the joint association of FI and OWT/OB on clinically measured hypertension. Among the few studies that exist, the combination of FI and obesity has been associated with greater postpartum weight gain (38) and worse mental health among women (22). In general, among women who experienced both FI and OWT/OB, higher odds of hypertension may be due to the stress of FI interacting with metabolic factors related to adiposity such as insulin resistance, inflammation, elevated cortisol levels, and other metabolic dysregulations (18). Nevertheless, we did not observe statistically significant associations with pre-hypertension, likely due to small subgroup sizes and limited statistical power.

Our findings highlight the importance of strengthening integrated nutrition and chronic disease prevention efforts in Guatemala, particularly within the framework of the National Food and Nutrition Security Policy 2022–2037 (Política Nacional de Seguridad Alimentaria y Nutricional; POLSAN), which incorporates prevention of overweight, obesity, and other noncommunicable diseases into the national food security agenda (54). Recent national initiatives under POLSAN have emphasized early detection of obesity, nutrition education, physical activity promotion, and multisectoral actions to address the double burden of malnutrition (55). Integrated policies addressing both nutritional vulnerability and chronic disease risk factors may help reduce future cardiovascular disease burden among Guatemalan women. Although the SIVESNU 2018–2019 cycle was the most recent publicly available nationally representative dataset at the time of analysis, recently released summaries from the 2022–2023 SIVESNU cycle suggest that OWT/OB among Guatemalan WRA continue to rise, indicating that the burden observed in our study likely remains highly relevant (56). Future studies should examine whether the joint association between FI, OWT/OB, and hypertension persists in more recent nationally representative data as these datasets become publicly available.

One of the key strengths of our study is its novelty in the Latin American context, addressing a critical gap on the intersection of food insecurity, obesity and hypertension (14). Using a nationally representative sample of WRA—a population highly vulnerable to nutrition and health disparities—including Indigenous and non-Indigenous women from both rural and urban areas, we ensured internal generalizability across different socioeconomic and geographic contexts of Guatemala. Additionally, data were collected using rigorous protocols for clinical measurements on weight and hypertension, minimizing bias associated with self-reported data (57). FI was assessed using the FIES scale, a standardized and validated tool appropriate for the regional context (33). We were also able to account for both individual and household confounders, including SEP which we derived using PCA, a robust and validated methodology used in previous studies in the absence of income data.

Our study is not absent of limitations. As a cross-sectional study, it cannot establish temporal order or causality, particularly given the complexity of the relationship under study. Additionally, information bias may be present as BMI was used to classify obesity instead of more precise measures of adiposity such as bioelectrical impedance analysis or dual-energy X-ray absorptiometry (58). This may have led to misclassification, likely nondifferential, potentially attenuating the observed associations. While there is no gold standard for measuring FI, capturing it at the household level may not fully reflect its severity at the individual level due to intra-household food allocation differences and is subject to recall and social desirability bias (14), which could either overestimate or underestimate the prevalence and its association with hypertension. Residual confounding is also a concern, as neighborhood-level data and other relevant individual factors, such as marital status and dietary intake, were not available; this could bias estimates in either direction depending on the distribution of these unmeasured factors. Finally, SIVESNU sampling and the specific sociocultural and economic context of Guatemala may limit the generalizability of our findings to WRA living in households without young children, older women >49 years old, men, and other populations of high-income countries or with less ethnic diversity. However, given the absence of national estimates on this topic in Guatemala prior to our study, our findings have the potential to inform national policy and program design aimed at addressing the interplay between FI, obesity, and hypertension.

## 5 Conclusion

With nearly 40% of Guatemalan WRA having high blood pressure, those with OWT/OB, with or without FI, had higher odds of hypertension compared to those without OWT/OB and FI. Although the coexistence of FI and OWT/OB was significantly associated with hypertension, OWT/OB alone showed the strongest associations, suggesting that excess body weight may be the primary driver of hypertension risk in this population. Patterns of FI and OWT/OB differed substantially by SEP status and urbanicity. Socioeconomic disparities in malnutrition and hypertension highlight the need for targeted interventions addressing this coexisting burden and the urgency of policies that improve food access and promote healthier dietary patterns. Future research should explore longitudinal associations to better understand underlying mechanisms.

## Data Availability

Publicly available datasets were analyzed in this study. This data can be found at: Health and Nutrition Epidemiological Surveillance System Survey (SIVESNU)/Secretariat of Food Security and Nutrition, Presidency of Guatemala (SIINSAN) at https://portal.siinsan.gob.gt/monitoreo-y-evaluacion/#1643404186375-f4d98353-dd51.

## References

[1] FAO, IFAD, UNICEF, WFP, WHO. The State of Food Security and Nutrition in the World 2024.FAO; IFAD; UNICEF; WFP; WHO. Rome. (2024).

[2] World Bank. What Is Food Security? There Are Four Dimensions. (2026) Available online at: https://www.worldbank.org/en/topic/agriculture/brief/food-security-update/what-is-food-security (Accessed October 26, 2023)

[3] U.S. Agency for International Development (USAID). Food Security Guatemala | U.S. Agency for International Development. (2022). Available online at: https://www.usaid.gov/guatemala/our-work/food-security (Accessed November 8, 2023)

[4] Christian TJ. Associations with urban sprawl, food insecurity, and the joint insecurity-obesity paradox. SSRN Electron J. (2009). doi: 10.2139/SSRN.1491754

[5] Dhurandhar EJ. The food-insecurity obesity paradox: a resource scarcity hypothesis. Physiol Behav. (2016) 162:88–92. doi: 10.1016/J.PHYSBEH.2016.04.025, 27126969 PMC5394740

[6] Stuff JE Casey PH Connell CL Champagne CM Gossett JM Harsha D . Household food insecurity and obesity, chronic disease, and chronic disease risk factors. J Hunger Environ Nutr. (2007) 1:43–62. doi: 10.1300/J477V01N02_04

[7] Carvajal-Aldaz D Cucalon G Ordonez C. Food insecurity as a risk factor for obesity: a review. Front Nutr. (2022) 9:1012734. doi: 10.3389/fnut.2022.1012734, 36225872 PMC9549066

[8] Frongillo EA Bernal J. Understanding the coexistence of food insecurity and obesity. Curr Pediatr Rep. (2014) 2:284–90. doi: 10.1007/s40124-014-0056-6

[9] López-Cepero A Frisard C Bey G Lemon SC Rosal MC. Association between food insecurity and emotional eating in Latinos and the mediating role of perceived stress. Public Health Nutr. (2019) 23:642–8. doi: 10.1017/S1368980019002878, 31718732 PMC7060105

[10] Rodríguez LA Mundo-Rosas V Méndez-Gómez-Humarán I Pérez-Escamilla R Shamah-Levy T. Dietary quality and household food insecurity among Mexican children and adolescents. Matern Child Nutr. (2017) 13:e12372. doi: 10.1111/MCN.12372, 27863001 PMC6866226

[11] Rutten LJF Yaroch AL Colón-Ramos U Johnson-Askew W Story M. Poverty, food insecurity, and obesity: a conceptual framework for research, practice, and policy. J Hunger Environ Nutr. (2010) 5:403–15. doi: 10.1080/19320248.2010.527275

[12] Taylor EA Foster JS Mobley AR. Examining factors related to the food insecurity–obesity paradox in low-income mothers and fathers. Food Nutr Bull (2021) 42:309–16. doi: 10.1177/03795721211011133, 34002624

[13] Kowaleski-Jones L Wen M Fan JX. Unpacking the paradox: testing for mechanisms in the food insecurity and BMI association. J Hunger Environ Nutr. (2019) 14:683–97. doi: 10.1080/19320248.2018.1464997

[14] Brown AGM Esposito LE Fisher RA Nicastro HL Tabor DC Walker JR. Food insecurity and obesity: research gaps, opportunities, and challenges. Transl Behav Med. (2019) 9:980–7. doi: 10.1093/TBM/IBZ117, 31570918 PMC6937550

[15] Briggs R Rowden H Lagojda L Robbins T Randeva HS. The lived experience of food insecurity among adults with obesity: a quantitative and qualitative systematic review. J Public Health. (2024) 46:230–49. doi: 10.1093/PUBMED/FDAE016, 38409966 PMC11141780

[16] Beltrán S Pharel M Montgomery CT López-Hinojosa IJ Arenas DJ DeLisser HM. Food insecurity and hypertension: a systematic review and meta-analysis. PLoS One. (2020) 15:e0241628. doi: 10.1371/JOURNAL.PONE.0241628, 33201873 PMC7671545

[17] Irving SM Njai RS Siegel PZ. Peer reviewed: food insecurity and self-reported hypertension among Hispanic, Black, and white adults in 12 states, Behavioral Risk Factor Surveillance System, 2009. Prev Chronic Dis. (2014) 11:140190. doi: 10.5888/PCD11.140190, 25232748 PMC4170725

[18] Patel SA Ali MK Alam D Yan LL Levitt NS Bernabe-Ortiz A . Obesity and its relation with diabetes and hypertension: a cross-sectional study across 4 geographical regions. Glob Heart. (2016) 11:71–79.e4. doi: 10.1016/J.GHEART.2016.01.003, 27102024 PMC4843822

[19] Bonavire K Williams G Sastre L. P128 diet quality, food access, obesity and hypertension risk in rural Nicaragua. J Nutr Educ Behav. (2019) 51:S90. doi: 10.1016/J.JNEB.2019.05.504

[20] Miranda JJ Herrera VM Chirinos JA Gomez LG Perel P Pichardo R . Major cardiovascular risk factors in Latin America: a comparison with the United States. The Latin American Consortium of Studies in Obesity (LASO). PLoS One. (2013) 8:e54056. doi: 10.1371/JOURNAL.PONE.0054056 23349785 PMC3547948

[21] Del Rio AI Velásquez IM Roa R Mendoza RM Motta J Quintana HK. Prevalence of hypertension and possible risk factors of hypertension unawareness among individuals aged 30–75 years from two Panamanian provinces: results from population-based cross-sectional studies, 2010 and 2019. PLoS One. (2022) 17:e0276222. doi: 10.1371/JOURNAL.PONE.0276222, 36441768 PMC9704556

[22] Martínez-Jaikel T Frongillo EA. Primary role of discouragement in coexistence of food insecurity and excess weight in Costa Rican women. J Hunger Environ Nutr. (2016) 11:210–26. doi: 10.1080/19320248.2016.1157546

[23] Integrated Food Security Phase Clasification (IPC). Guatemala Acute Food Insecurity Situation March to May 2022 and Projections for June–September 2022 and October 2022–February 2023 | IPC - Integrated Food Security Phase Classification. Guatemala City. (2022). Available online at: https://www.ipcinfo.org/ipc-country-analysis/details-map/en/c/1155665/?iso3=GTM (Accessed February 4, 2025)

[24] World Health Organization (WHO). Guatemala - Health data Overview (2022). Available online at: https://data.who.int/countries/320 (Accessed June 30, 2024)

[25] Mazariegos M Kroker-Lobos MF Ramírez-Zea M. Socio-economic and ethnic disparities of malnutrition in all its forms in Guatemala. Public Health Nutr. (2020) 23:s68–76. doi: 10.1017/S1368980019002738, 31588883 PMC10200633

[26] Ramirez-Zea M Kroker-Lobos MF Close-Fernandez R Kanter R. The double burden of malnutrition in indigenous and nonindigenous Guatemalan populations. Am J Clin Nutr. (2014) 100:1644S–51S. doi: 10.3945/ajcn.114.083857, 25411307

[27] Rosenthal J Lopez-Pazos E Dowling NF Pfeiffer CM Mulinare J Vellozzi C . Folate and vitamin B12 deficiency among non-pregnant women of childbearing-age in Guatemala 2009–2010: prevalence and identification of vulnerable populations. Matern Child Health J. (2015) 19:2272–85. doi: 10.1007/s10995-015-1746-6, 26002178 PMC4575840

[28] Pickens CM Flores-Ayala R Addo OY Whitehead RD Jr Palmieri M Ramirez-Zea M . Prevalence and predictors of high blood pressure among women of reproductive age and children aged 10 to 14 years in Guatemala. Prev Chronic Dis. (2020) 17:E66. doi: 10.5888/PCD17.190403, 32701434 PMC7380295

[29] Herrera-Cuenca M Previdelli AN Koletzko B Hernandez P Landaeta-Jimenez M Sifontes Y . Childbearing age women characteristics in Latin America. Building evidence bases for early prevention. Results from the ELANS study. Nutrients. (2021) 13:45. doi: 10.3390/NU13010045 PMC7824015 33375712

[30] Black RE Allen LH Bhutta ZA Caulfield LE de Onis M Ezzati M . Maternal and child undernutrition: global and regional exposures and health consequences. Lancet. (2008) 371:243–60. doi: 10.1016/S0140-6736(07)61690-0, 18207566

[31] Palmieri M Flores-Ayala R Mesarina K Mazariegos DI Martínez C López B . Experiences and lessons learned in developing and implementing a population-based nutrition and health surveillance system in Guatemala 2011-2021. Curr Dev Nutr. (2022) 6:nzac027. doi: 10.1093/CDN/NZAC027, 35475139 PMC9022727

[32] Food Insecurity Experience Scale (FIES). INDDEX Project. (2023). Available online at: https://inddex.nutrition.tufts.edu/data4diets/indicator/food-insecurity-experience-scale-fies (Accessed November 10, 2024)

[33] Latin American and Caribbean Food Security Scale (ELCSA) | INDDEX Project. (2023). Available online at: https://inddex.nutrition.tufts.edu/data4diets/indicator/latin-american-and-caribbean-food-security-scale-elcsa (Accessed April 10, 2024)

[34] Segall Corrêa AM Álvarez Uribe MC Melgar Quiñonez H. Pérez Escamilla R. Escala latinoamericana y caribeña de seguridad alimentaria (Elcsa): manual de uso y aplicaciones. Roma: FAO (2012): 78. Available online at: https://bibliotecadigital.udea.edu.co/handle/10495/25324 (Accessed April 10, 2024)

[35] Ballard TJ Kepple AW Cafiero CD. The food insecurity experience scale (2013). Rome: Food and Agriculture Organization of the United Nations. Available online at: https://openknowledge.fao.org/handle/20.500.14283/as583e (Accessed March 16, 2025)

[36] Cafiero C Viviani S Nord M. Food security measurement in a global context: the food insecurity experience scale. Measurement. (2018) 116:146–52. doi: 10.1016/J.MEASUREMENT.2017.10.065

[37] Whelton PK Carey RM Aronow WS Casey DE Jr Collins KJ Fennison Himmelfarb C. 2017 ACC/AHA/AAPA/ABC/ACPM/AGS/APhA/ASH/ASPC/NMA/PCNA Guideline for the Prevention, Detection, Evaluation, and Management of High Blood Pressure in Adults: a report of the American College of Cardiology/American Heart Association Task Force on Clinical Practice Guidelines. J Am Coll Cardiol. (2018) 71:e127–248. doi: 10.1016/J.JACC.2017.11.006 29146535

[38] Olson CM Strawderman MS. The relationship between food insecurity and obesity in rural childbearing women. J Rural Health. (2008) 24:60–6. doi: 10.1111/J.1748-0361.2008.00138.X, 18257872

[39] Zhu Y Wang Z. Association between joint physical activity and healthy dietary patterns and hypertension in US adults: cross-sectional NHANES study. BMC Public Health. (2024) 24:1–9. doi: 10.1186/s12889-024-18346-8 38504199 PMC10953194

[40] Te Vazquez J Feng SN Orr CJ Berkowitz SA. Food insecurity and cardiometabolic conditions: a review of recent research. Curr Nutr Rep. (2021) 10:243–54. doi: 10.1007/s13668-021-00364-2, 34152581 PMC8216092

[41] Castillo DC Ramsey NLM Yu SSK Ricks M Courville AB Sumner AE. Inconsistent access to food and cardiometabolic disease: the effect of food insecurity. Curr Cardiovasc Risk Rep. (2012) 6:245–50. doi: 10.1007/s12170-012-0236-2, 22629473 PMC3357002

[42] World Health Organization (WHO). Global Physical Activity Questionnaire (GPAQ). (2021). Available online at: https://www.who.int/publications/m/item/global-physical-activity-questionnaire (Accessed November 25, 2024)

[43] Auchincloss AH Michael YL Niamatullah S Li S Melly SJ Pharis ML . Changes in physical activity after joining a bikeshare program: a cohort of new bikeshare users. Int J Behav Nutr Phys Act. (2022) 19:1–14. doi: 10.1186/s12966-022-01353-6 36195957 PMC9533574

[44] Guatemala’s National Institute of Statistics. Censo de Población y Vivienda 2018. Guatemala City: Informe General (2018).

[45] Vyas S Kumaranayake L. Constructing socio-economic status indices: how to use principal components analysis. Health Policy Plan. (2006) 21:459–68. doi: 10.1093/HEAPOL/CZL029, 17030551

[46] 2024 ESC Guidelines for Management of Elevated BP and Hypertension: Key Points. London: American College of Cardiology. (2024). Available online at: https://www.acc.org/Latest-in-Cardiology/ten-points-to-remember/2024/09/05/14/11/2024-esc-guidelines-for-bp-esc-2024 (Accessed December 2, 2024)

[47] James PA Oparil S Carter BL Cushman WC Dennison-Himmelfarb C Handler J . 2014 Evidence-Based Guideline for the Management of High Blood Pressure in Adults: Report from the Panel Members Appointed to the Eighth Joint National Committee (JNC 8). JAMA. (2014) 311:507–20. doi: 10.1001/JAMA.2013.284427, 24352797

[48] Salehi-Abargouei A Maghsoudi Z Shirani F Azadbakht L. Effects of dietary approaches to stop hypertension (DASH)-style diet on fatal or nonfatal cardiovascular diseases-incidence: a systematic review and meta-analysis on observational prospective studies. Nutrition. (2013) 29:611–8. doi: 10.1016/j.nut.2012.12.018, 23466047

[49] Iannotti LL Robles M Pachón H Chiarella C. Food prices and poverty negatively affect micronutrient intakes in Guatemala. J Nutr. (2012) 142:1568–76. doi: 10.3945/JN.111.157321, 22695968

[50] Beveridge L Whitfield S Fraval S Van Wijk M Van Etten J Mercado L . Experiences and drivers of food insecurity in Guatemala’s dry corridor: insights from the integration of ethnographic and household survey data. Front Sustain Food Syst. (2019) 3:464185. doi: 10.3389/FSUFS.2019.00065/BIBTEX

[51] Ford ND Martorell R Ramirez-Zea M Stein AD. The nutrition transition in rural Guatemala: 12 year changes in diet of adults. FASEB J. (2018) 31:147.3–3. doi: 10.1096/FASEBJ.31.1_SUPPLEMENT.147.3

[52] Ashe KM Lapane KL. Food insecurity and obesity: exploring the role of social support. J Womens Health (Larchmt) (2018) 27:651–8. doi: 10.1089/JWH.2017.6454, 29182494

[53] Hernández-Ruiz Á Madrigal C Soto-Méndez MJ Gil Á. Challenges and perspectives of the double burden of malnutrition in Latin America. Clínica e Investigación en Arteriosclerosis (English Edition). (2022) 34:3–16. doi: 10.1016/j.arteri.2021.11.005 35153111

[54] Secretaría de Seguridad Alimentaria y Nutricional de la Presidencia de la República (SESAN) de Guatemala. Política Nacional de Seguridad Alimentaria y Nutricional. 1st edn. Guatemala City. 84 pages. (2023).

[55] Secretaría de Seguridad Alimentaria y Nutricional de la Presidencia de la República (SESAN) de Guatemala. Las 4 acciones contra la obesidad y sobrepeso impulsadas en la Política Nacional de Seguridad Alimentaria y Nutricional – SESAN Guatemala. (2023). Available online at: https://portal.sesan.gob.gt/2023/03/02/las-4-acciones-contra-la-obesidad-y-sobrepeso-impulsadas-en-la-politica-nacional-de-seguridad-alimentaria-y-nutricional/ (Accessed May 18, 2026)

[56] Secretaría de Seguridad Alimentaria y Nutricional de la Presidencia de la República (SESAN) de Guatemala. Guatemala enfrenta una doble crisis nutricional: desnutrición crónica y sobrepeso y obesidad – SESAN Guatemala. Guatemala City. (2026). Available online at: https://portal.sesan.gob.gt/2026/03/13/guatemala-enfrenta-una-doble-crisis-nutricional-desnutricion-cronica-y-sobrepeso-y-obesidad/?utm_source=chatgpt.com (Accessed May 18, 2026)

[57] Gonçalves VSS Andrade KRC Carvalho KMB Silva MT Pereira MG Galvao TF. Accuracy of self-reported hypertension: a systematic review and meta-analysis. J Hypertens. (2018) 36:970–8. doi: 10.1097/HJH.0000000000001648, 29232280

[58] Okorodudu DO Jumean MF Montori VM Romero-Corral A Somers VK Erwin PJ . Diagnostic performance of body mass index to identify obesity as defined by body adiposity: a systematic review and meta-analysis. Int J Obes. (2010) 34:791–9. doi: 10.1038/ijo.2010.5, 20125098

[59] Beltrán S Pharel M Montgomery CT López-Hinojosa IJ Arenas DJ DeLisser HM. Food insecurity and hypertension: A systematic review and meta-analysis. PLoS One. (2020) 15:e0241628. doi: 10.1371/JOURNAL.PONE.0241628 33201873 PMC7671545

