# Supplementary material for: Hypertension among Guatemalan women: the role of food insecurity and overweight/obesity
Source: Front Nutr. 2026 Jun 16;13:1857055. doi: 10.3389/fnut.2026.1857055 (PMC13339682; doi:10.3389/fnut.2026.1857055)
Supplement: Supplementary file 1 [file Table_1.DOCX]

# **Figure 1:** Flowchart of sample selection

**SIVESNU 2018/19**

1,755 WRA

**Ineligible for present study**

99 Pregnant women and those with missing pregnancy status

**Eligible participants**

1,656 WRA (100%)

118 WRA with missing data on key variables

**Final sample**

1,538 WRA (93%)

# **Supplement Table 1:** Excluded vs Included Participants comparison

|  | TOTAL | | Included | | Excluded | |
| --- | --- | --- | --- | --- | --- | --- |
| Variables | **N** | **col %** | **N** | **col %** | **N** | **col %** |
| TOTAL | 1656 | 100% | 1538 | 93% | 118 | 7% |
| Hypertension status |  |  |  |  |  |  |
| Missing | 81 | 5% |  |  | 81 | 76% |
| Normal | 971 | 59% | 946 | 62% | 25 | 21% |
| Pre-HTN | 432 | 26% | 100 | 7% | 1 | 1% |
| HTN | 172 | 10% | 492 | 32% | 11 | 9% |
| Food Insecurity (FI) |  |  |  |  |  |  |
| No FI (0) | 434 | 26% | 366 | 27% | 68 | 31% |
| Mild (1-3) | 653 | 39% | 538 | 40% | 115 | 35% |
| Moderate (4-6) | 393 | 24% | 306 | 23% | 87 | 25% |
| Severe (7-8) | 176 | 11% | 132 | 10% | 44 | 9% |
| Overweight/obesity (OWT/OB) |  |  |  |  |  |  |
| Missing | 72 | 3% |  |  | 72 | 29% |
| Healthy weight | 632 | 29% | 611 | 40% | 21 | 18% |
| Overweight | 538 | 25% | 523 | 34% | 15 | 13% |
| Obese | 414 | 19% | 404 | 26% | 10 | 8% |
| Coexisting burden (FI + OWT/OB) |  |  |  |  |  |  |
| Missing | 72 | 4% |  |  | 72 | 61% |
| None | 153 | 9% | 144 | 9% | 9 | 8% |
| Just FI | 479 | 29% | 467 | 30% | 12 | 10% |
| Just OWT/OB | 258 | 16% | 253 | 16% | 5 | 4% |
| FI + OWT/OB | 694 | 42% | 674 | 44% | 20 | 17% |
| Age |  |  |  |  |  |  |
| 15-19 years | 251 | 15% | 240 | 16% | 11 | 9% |
| 20-29 years | 567 | 34% | 511 | 33% | 56 | 47% |
| 30-39 years | 510 | 31% | 480 | 31% | 30 | 25% |
| 40-49 years | 328 | 20% | 307 | 20% | 21 | 18% |
| Physical activity level |  |  |  |  |  |  |
| Missing | 11 | 1% |  |  | 11 | 9% |
| Sufficient >=150min/week | 710 | 43% | 672 | 44% | 38 | 32% |
| Insufficient <150/week | 935 | 56% | 866 | 56% | 69 | 59% |
| Lactating status |  |  |  |  |  |  |
| Not pregnant | 1377 | 83% | 1292 | 84% | 85 | 72% |
| Not pregnant, lactating | 279 | 17% | 246 | 16% | 33 | 28% |
| Socioeconomic position (SEP) |  |  |  |  |  |  |
| Low (0-1 components) | 584 | 35% | 533 | 35% | 51 | 43% |
| Middle (2-3 components) | 559 | 34% | 526 | 34% | 33 | 28% |
| High (4-9 components) | 513 | 31% | 479 | 31% | 34 | 29% |
| Total children in the household |  |  |  |  |  |  |
| 0 children | 181 | 11% | 171 | 11% | 10 | 8% |
| 1-2 children | 849 | 51% | 790 | 51% | 59 | 50% |
| >=3 children | 626 | 38% | 577 | 38% | 49 | 42% |
| Total adults in the household |  |  |  |  |  |  |
| Missing | 1 | 0% |  |  | 1 | 1% |
| 1-2 adults | 902 | 54% | 841 | 55% | 61 | 75% |
| 3-4 adults | 518 | 31% | 479 | 31% | 17 | 21% |
| >=5 adults | 235 | 14% | 218 | 14% | 2 | 2% |
| Area |  |  |  |  |  |  |
| Missing | 5 | 0% |  |  | 5 | 4% |
| Urban | 632 | 38% | 583 | 38% | 49 | 42% |
| Rural | 1019 | 62% | 955 | 62% | 64 | 54% |

# **Supplement Table 2:** Food Insecurity Scale (FIES) questionnaire^a^

|  |  | **Questionnaire** | |
| --- | --- | --- | --- |
|  |  | **Response options No or Yes** | |
|  |  | **English** | **Spanish** |
| # | **Short description** | ***Food security*** | ***Seguridad alimentaria*** |
| 1 | Worried about not having enough food to eat | During the previous 12 months, was there a time when you or others in your household worried about not having enough food to eat because of a lack of money or other resources? | Durante los últimos 12 MESES ha habido algún momento en que ¿Usted u otra persona en su hogar se haya preocupado por no tener suficientes alimentos para comer por falta de dinero? |
| 2 | Unable to eat healthy and nutritious food | During the previous 12 months, was there a time when you or others in your household were unable to eat healthy and nutritious food because of a lack of money or other resources? | Durante los últimos 12 MESES ha habido algún momento en que ¿hubo alguna vez en que usted u otra persona en su hogar no haya podido comer alimentos saludables y nutritivos por falta de dinero u otros recursos? |
| 3 | Ate only a few kinds of foods | During the previous 12 months, was there a time when you or others in your household ate only a few kinds of foods because of a lack of money or other resources? | Durante los últimos 12 MESES ha habido algún momento en que ¿Hubo alguna vez en que usted u otra persona en su hogar haya comido poca variedad de alimentos por falta de dinero u otros recursos? |
| 4 | Skipped a meal | During the previous 12 months, was there a time when you or others in your household had to skip a meal because there was not enough money or other resources to get food? | Durante los últimos 12 MESES ha habido algún momento en que ¿Hubo alguna vez en que usted u otra persona en su hogar haya tenido que dejar de desayudar, almorzar o cenar porque no había suficiente dinero u otros recursos para obtener alimentos? |
| 5 | Ate less | During the previous 12 months, was there a time when you or others in your household ate less than you thought you should because of a lack of money or other resources? | Durante los últimos 12 MESES ha habido algún momento en que ¿hubo alguna vez en que usted u otra persona en su hogar haya comido menos de lo que pensaba que debía comer por falta de dinero u otros recursos? |
| 6 | Ran out of food | During the previous 12 months, was there a time when your household ran out of food because of a lack of money or other resources? | Durante los últimos 12 MESES ha habido algún momento en que ¿Hubo alguna vez en que su hogar se haya quedado sin alimentos por falta de dinero u otros recursos? |
| 7 | Hungry but did not eat | During the previous 12 months, was there a time when you or others in your household were hungry but did not eat because there was not enough money or other resources for food? | Durante los últimos 12 MESES ha habido algún momento en que ¿Hubo alguna vez en que usted u otra persona en su hogar haya sentido hambre pero no comió porque no había suficiente dinero u otros recursos para obtener alimentos? |
| 8 | Went without eating for a whole day | During the previous 12 months, was there a time when you or others in your household went without eating for a whole day because of a lack of money or other resources? | Durante los últimos 12 MESES ha habido algún momento en que ¿Hubo alguna vez en que usted u otra persona en su hogar haya dejado de comer todo un día por falta de dinero u otros recursos? |
| *Abbreviations: FIES=Food Insecurity Scale.  ^a^Food and Agricultural Organization of the United Nations, 2016. Methods for Estimating Comparable Rates of Food Insecurity Experienced | | | |
| by Adults Throughout the World (Food Insecurity Experience Scale). | | | |
| URL: Https://Openknowledge.Fao.Org/Server/Api/Core/Bitstreams/823b2fcd-Bc52-4371-A31d-Ea2aa74e63d5/Content | | | |

# **Supplement Table 3:** Socioeconomic position (SEP) scale: list of variables and results from principal components analysis

| **Selected for inclusion** |  |  |  | **Rotated factor pattern**^b^ | |
| --- | --- | --- | --- | --- | --- |
| **in the scale**^a^ | **#** | **Item** | **% With this item** | **Factor #1**^c^ | **Factor #2** |
| Yes | 1 | Owns a TV | 75% | **0.88** | -0.11 |
| Yes | 2 | Owns a refrigerator | 49% | **0.87** | 0.00 |
| Yes | 3 | Owns a washing machine | 14% | **0.86** | 0.07 |
| Yes | 4 | Owns a landline phone at home | 6% | **0.84** | -0.05 |
| Yes | 5 | Owns a computer | 14% | **0.80** | 0.11 |
| Yes | 6 | Owns a microwave | 21% | **0.80** | 0.08 |
| Yes | 7 | Owns a vehicle for transportation^d^ | 37% | **0.71** | 0.13 |
| Yes | 8 | Participant has health insurance^e^ | 13% | **0.65** | -0.24 |
| Yes | 9 | Participant has ever gone to the dentist | 35% | **0.51** | 0.01 |
| No | 10 | Property is owned vs. rented/borrowed | 75% | -0.01 | **0.77** |
| No | 11 | N children living in the household (reversed direction, categorical) | 74% | 0.30 | -0.38 |
| No | 12 | More favorable quality of home environment^f^ | 87% | 0.29 | **0.61** |
|  |  |  |  |  |  |

^a^We began with 12 variables that were relevant to socioeconomic status. We reversed the direction of item 11 (above) so that higher values represented higher SEP. The 12 items were binary or ordinal, thus to execute a principal components analysis (PCA), we first derived a polychoric correlation matrix and then used the matrix as input data for the PCA. We used rotation (varimax rotation) to improve interpretability of the factor loadings.

^b^Factor 1 and factor 2 accounted for 47% and 10% of the total variance, All retained items were identified in Factor 1 and had loadings of at least ≥0.50. Factor 2 was not used because it explained a low % of variance and only had 2 variables that loaded highly on it.

^c^All 9 of the retained items were binary variables (0,1). We summed up the 9 items to derive a score and then classified the score in tertiles, assigning tertile 1 (lower SEP, score 0-1) tertile 2 (middle SEP, score 2-3) and tertile 3 (higher SEP, score 4-9). Nine out of 12 items were retained and subsequently used in a scale to proxy "socioeconomic position".

^d^Vehicle for transportation was derived from affirmative responses to some member of the household "having and using for transportation"

any of the following: motorcycle, car, van, pick-up truck, large truck.

^e^Has private health insurance or social security insurance.

^f^More favorable quality of home environment was assessed by questions that affirmed that household sleeping space was separate from cooking space.

# **Supplement Table 4:** Adjusted association between food insecurity and OWT/OB status, and alternative cutoffs for hypertension (N= 1538)

|  | **JNC-8**† | | | | **ESC-2024** | | | |
| --- | --- | --- | --- | --- | --- | --- | --- | --- |
|  | **Pre-hypertensive vs. normotensive**† | | **Hypertensive vs. normotensive** | | **Pre-hypertensive vs. normotensive**† | | **Hypertensive vs. normotensive** | |
|  | OR (95% CI)* | P-value | OR (95% CI) | P-value | OR (95% CI) | P-value | OR (95% CI) | P-value |
| **Model 1:** Unadjusted | | | | | | |  |  |
| Neither | *Referent* |  | *Referent* |  | *Referent* |  | *Referent* |  |
| Only FI | 0.88 (0.52, 1.47) | 0.610 | 1.22 (0.46, 3.21) | 0.684 | 0.17 (0.81, 1.69) | 0.404 | 1.37 (0.13, 3.67) | 0.526 |
| Only OWT/OB | 2.70 (1.42, 5.12) | **0.003** | 5.46 (1.81, 16.48) | **0.003** | 3.14 (1.89, 5.20) | **<0.0001** | 8.59 (2.26, 27.38) | **0.0004** |
| Coexisting burden | 2.11 (1.24, 3.60) | **0.007** | 5.75 (2.03, 16.31) | **0.001** | 2.91 (1.04, 4.40) | **<0.0001** | 9.44 (3.17, 28.14) | **<0.0001** |
| **Model 2:** Adjusted for individual-level covariates‡ | | | | | | |  |  |
| Neither | *Referent* |  | *Referent* |  | *Referent* |  | *Referent* |  |
| Only FI | 0.92 (0.53, 1.59) | 0.757 | 1.13 (0.40, 3.20) | 0.813 | 1.17 (0.81, 1.69) | 0.406 | 1.26 (0.44, 3.61) | 0.662 |
| Only OWT/OB | 2.35 (1.23, 4.48) | **0.010** | 3.76 (1.28, 11.04) | **0.017** | 2.57 (1.55, 4.27) | **0.0004** | 5.21 (1.71, 16.48) | **0.004** |
| Coexisting burden | 1.63 (0.94, 2.81) | 0.083 | 3.13 (1.11, 8.80) | **0.031** | 2.16 (1.41, 3.30) | **0.001** | 4.55 (1.54, 13.38) | **0.007** |
| **Model 3:** Adjusted for Model 2 covariates + household-level covariates§ | | | | | | |  |  |
| Neither | *Referent* |  | *Referent* |  | *Referent* |  | *Referent* |  |
| Only FI | 0.83 (0.47, 1.46) | 0.516 | 1.10 (0.38, 0.86) | 0.694 | 1.12 (0.78, 1.62) | 0.538 | 1.24 (0.42, 3.68) | 0.694 |
| Only OWT/OB | 2.48 (1.29, 4.76) | **0.007** | 3.95 (1.39, 11.23) | **0.011** | 2.74 (1.64, 4.56) | **0.0002** | 5.78 (1.92, 17.44) | **0.002** |
| Coexisting burden | 1.53 (0.87, 2.66) | 0.136 | 3.03 (1.05, 8.75) | **0.041** | 2.09 (1.36, 3.22) | **0.001** | 4.42 (1.45, 13.40) | **0.009** |
| **Model 3b:** Variation of Model 3 | | | | | | | | |
| Neither | *Referent* |  | *Referent* |  | *Referent* |  | *Referent* |  |
| Only FI | 0.80 (0.46, 1.38) | 0.411 | 1.04 (0.35, 3.03) | 0.949 | 1.11 (0.77, 1.61) | 0.575 | 1.18 (0.40, 3.51) | 0.768 |
| Only OWT/OB | 2.58 (1.34, 4.99) | **0.005** | 4.01 (1.40, 11.49) | **0.010** | 2.77 (1.67, 4.60) | **0.0001** | 5.82 (1.92, 17.76) | **0.002** |
| Coexisting burden | 1.56 (0.90, 2.72) | 0.113 | 3.03 (1.04, 8.88) | **0.043** | 2.13 (1.39, 3.29) | **0.001** | 4.46 (1.45, 13.74) | **0.010** |

All multinomial logistic regression analysis accounted for survey weights. **P-value** ≤0.05 is considered statistically significant.

*Abbreviations: OR=odds ratio. CI=confidence interval. FI=food insecurity. OWT/OB=overweight/obesity. SBP=systolic blood pressure. DBP=diastolic blood pressure. JNC=Joint National Committee. ESC=European Society of Cardiology.

†Hypertension status was defined as: 1)JNC-8 guidelines (pre-hypertension 120-139 and DBP 80-89, hypertension SBP≥140 or DBP≥90); and 2)ESC-2024 guidelines (pre-hypertension 120-139 and DBP 70-89, hypertension SBP SBP≥140 or DPB≥90).

‡ Individual-level covariates: age group (15-19y, 20-29y, 30-39y, 40-49y), physical activity (tertiles of min/week), lactating status (yes/no).

§Household-level covariates: socio-economic position (tertiles), number of children in the household (0, 1-2, ≥3), number of adults in the household (1-2, 3-4, ≥5), area (urban vs rural).

¶ Includes same covariates as Model 2 + socio-economic position (tertiles), number of children birthed (0, 1-2, ≥3), number of adults in the household (1-2, 3-4, ≥5), area (urban vs rural).

|  |  |  | | |
| --- | --- | --- | --- | --- |
|  |  |  |  |  |
|  |  |  |  |  |
|  |  |  |  |  |
|  |  |  |  |  |
|  |  |  |  |  |
|  |  |  |  |  |
|  |  |  |  |  |
|  |  |  |  |  |
|  |  |  |  |  |
|  |  |  |  |  |
|  |  |  |  |  |
|  |  |  |  |  |
|  |  |  |  |  |
|  |  |  |  |  |
|  |  |  |  |  |
|  |  |  |  |  |
|  |  |  |  |  |
|  |  |  |  |  |
|  |  |  |  |  |
|  |  |  |  |  |
|  |  |  |  |  |
|  |  |  |  |  |
|  |  |  |  |  |
|  |  |  |  |  |
|  |  |  |  |  |
|  |  |  |  |  |
|  |  |  |  |  |
|  |  |  |  |  |
|  |  |  |  |  |
|  |  |  |  |  |
|  |  |  |  |  |
|  |  |  |  |  |
|  |  |  |  |  |
|  |  |  |  |  |
|  |  |  |  |  |
|  |  |  |  |  |
|  |  |  |  |  |
|  |  |  |  |  |
|  |  |  |  |  |
|  |  |  |  |  |
|  |  |  |  |  |
|  |  |  |  |  |
|  |  |  |  |  |
|  |  |  |  |  |
|  |  |  |  |  |
|  |  |  |  |  |

# **Supplement Table 5:** Adjusted association between food insecurity and OWT/OB status (decomposed in 8-categories), and outcomes for hypertension and systolic blood pressure (N= 1538)

|  | **Outcome 1: Hypertension (AHA/ACC) *** | | | | **Outcome 2: SBP (mm Hg, continuous) *** | | **Outcome 3: Hypertension (JNC-8)*** | | | | **Outcome 4: Hypertension (ESC 2024) *** | | | |
| --- | --- | --- | --- | --- | --- | --- | --- | --- | --- | --- | --- | --- | --- | --- |
|  | **Pre-hypertensive vs. normotensive** | | **Hypertensive vs. normotensive** | |  |  | **Pre-hypertensive vs. normotensive** | | **Hypertensive vs. normotensive** | | **Pre-hypertensive vs. normotensive** | | **Hypertensive vs. normotensive** | |
|  | OR (95% CI) | P-value | OR (95% CI) | P-value | β Coeff. | P-  value | OR (95% CI) | P-value | OR (95% CI) | P-value | OR (95% CI) | P-  value | OR (95% CI) | P  -value |
| **Model 1:** Unadjusted | | | | | | | | | | | | | | |
| Neither | Referent |  | Referent |  | Referent |  | Referent |  | Referent |  | Referent |  | Referent |  |
| Only Mild FI | 1.33 (0.35, 5.03) | 0.676 | 0.81 (0.43, 1.52) | 0.499 | 0.14 (-2.64, 2.92) | 0.921 | 0.88 (0.48, 1.62) | 0.681 | 1.01 (0.3, 3.36) | 0.988 | 1.14 (0.77, 1.69) | 0.497 | 1.12 (0.33, 3.77) | 0.856 |
| Only Moderate FI | 0.35 (0.34, 5.36) | 0.665 | 1.28 (0.74, 2.24) | 0.375 | 3.05 (-0.71, 6.81) | 0.111 | 1.11 (0.61, 2.01) | 0.741 | 2.23 (0.83, 6.03) | 0.113 | 1.49 (0.9, 2.46) | 0.117 | 2.76 (1, 7.67) | 0.051 |
| Only Severe FI | 0.86 (0.17, 4.45) | 0.860 | 0.4 (0.17, 0.95) | **0.038** | -0.42 (-3.93, 3.09) | 0.811 | 0.52 (0.22, 1.2) | 0.125 | 0.32 (0.06, 1.8) | 0.193 | 0.83 (0.45, 1.55) | 0.563 | 0.32 (0.06, 1.89) | 0.207 |
| Only OWT/OB | 2.89 (0.8, 10.43) | 0.104 | 3.23 (1.75, 5.96) | **0.0003** | 5.95 (2.95, 8.95) | **0.0002** | 2.7 (1.42, 5.13) | **0.003** | 5.47 (1.81, 16.56) | **0.003** | 3.14 (1.89, 5.21) | **<0.0001** | 8.59 (2.69, 27.5) | **0.0004** |
| Mild Coexisting burden | 1.86 (0.58, 5.9) | 0.291 | 3.02 (1.64, 5.54) | **0.001** | 6.89 (3.81, 9.99) | **<0.0001** | 2.27 (1.28, 4.02) | **0.006** | 5.42 (1.79, 16.44) | **0.003** | 2.93 (1.81, 4.74) | **<0.0001** | 8.67 (2.7, 27.82) | **0.0004** |
| Moderate Coexisting burden | 2.39 (0.7, 8.15) | 0.164 | 2.45 (1.38, 4.34) | **0.002** | 7.52 (4.27, 10.78) | **<0.0001** | 1.77 (1, 3.13) | 0.052 | 5.73 (2.02, 16.24) | **0.001** | 3.5 (2.03, 6.01) | **<0.0001** | 11.57 (3.64, 36.8) | **<0.0001** |
| Severe Coexisting burden | 1.7 (0.32, 9.12) | 0.532 | 3.56 (1.78, 7.12) | **0.0004** | 7.43 (3.4, 11.45) | **0.000** | 2.4 (1.21, 4.75) | **0.013** | 7.21 (2, 25.96) | **0.003** | 1.98 (1.08, 3.62) | **0.027** | 8.44 (2.21, 32.23) | **0.002** |
| **Model 2:** Adjusted for individual-level variables † | | | | | | | | | | | | | | |
| Neither | Referent |  | Referent |  | Referent |  | Referent |  | Referent |  | Referent |  | Referent |  |
| Only Mild FI | 1.43 (0.36, 5.17) | 0.612 | 0.8 (0.4, 1.6) | 0.524 | 0.2 (-2.58, 2.98) | 0.881 | 0.92 (0.48, 1.77) | 0.808 | 0.94 (0.26, 3.39) | 0.921 | 1.15 (0.77, 1.72) | 0.481 | 1.04 (0.28, 3.79) | 0.957 |
| Only Moderate FI | 1.38 (0.33, 5.72) | 0.659 | 1.26 (0.71, 2.26) | 0.427 | 2.79 (-1.09, 6.67) | 0.157 | 1.14 (0.61, 2.15) | 0.681 | 2.06 (0.72, 5.9) | 0.178 | 1.46 (0.88, 2.45) | 0.145 | 2.51 (0.85, 7.39) | 0.094 |
| Only Severe FI | 0.89 (0.16, 4.94) | 0.890 | 0.4 (0.17, 0.97) | **0.042** | -0.13 (-3.74, 3.48) | 0.943 | 0.55 (0.23, 1.29) | 0.167 | 0.29 (0.05, 1.73) | 0.172 | 0.83 (0.45, 1.53) | 0.542 | 0.29 (0.05, 1.79) | 0.180 |
| Only OWT/OB | 2.66 (0.73, 9.69) | 0.138 | 2.53 (1.36, 4.69) | **0.004** | 4.16 (1.23, 7.08) | **0.006** | 2.34 (1.23, 4.47) | **0.011** | 3.75 (1.27, 11.06) | **0.017** | 2.56 (1.54, 4.27) | **0.0004** | 5.29 (1.7, 16.49) | **0.005** |
| Mild Coexisting burden | 1.58 (0.5, 4.96) | 0.428 | 2.01 (1.08, 3.74) | **0.029** | 4.32 (1.39, 7.26) | **0.004** | 1.74 (0.97, 3.12) | 0.061 | 2.99 (1.00, 9.00) | 0.051 | 2.17 (1.31, 3.55) | **0.003** | 4.21 (1.34, 13.29) | **0.015** |
| Moderate Coexisting burden | 1.96 (0.58, 6.56) | 0.275 | 1.65 (0.9, 3.05) | 0.108 | 5.12 (1.98, 8.26) | **0.002** | 1.38 (0.75, 2.54) | 0.298 | 3.14 (1.13, 8.76) | **0.029** | 2.58 (1.48, 4.5) | **0.001** | 5.67 (1.79, 17.91) | **0.004** |
| Severe Coexisting burden | 1.42 (0.28, 7.29) | 0.671 | 2.2 (1.01, 4.78) | **0.046** | 4.35 (0.38, 8.32) | **0.032** | 1.77 (0.87, 3.62) | 0.115 | 3.55 (0.88, 14.23) | 0.074 | 1.42 (0.76, 2.66) | 0.269 | 3.56 (0.83, 15.15) | 0.086 |
| **Model 3a:** Adjusted for model 2 variables and household variables § | | | | | | | | | | | | | | |
| Neither | Referent |  | Referent |  | Referent |  | Referent |  | Referent |  | Referent |  | Referent |  |
| Only Mild FI | 1.3 (0.31, 2.88) | 0.722 | 0.77 (0.38, 1.56) | 0.456 | -0.34 (-3.16, 2.48) | 0.811 | 0.86 (0.44, 1.65) | 0.637 | 0.95 (0.26, 3.51) | 0.934 | 1.11 (0.74, 1.66) | 0.613 | 1.05 (0.28, 3.94) | 0.943 |
| Only Moderate FI | 1.29 (0.29, 3.83) | 0.741 | 1.15 (0.64, 2.09) | 0.633 | 2.07 (-1.82, 5.96) | 0.294 | 1.02 (0.54, 1.96) | 0.942 | 1.97 (0.64, 6.02) | 0.232 | 1.4 (0.85, 2.31) | 0.189 | 2.43 (0.78, 7.58) | 0.125 |
| Only Severe FI | 0.84 (0.14, 2.91) | 0.847 | 0.34 (0.14, 0.82) | **0.018** | -1.01 (-4.71, 2.69) | 0.590 | 0.45 (0.19, 1.09) | 0.076 | 0.27 (0.05, 1.62) | 0.150 | 0.76 (0.4, 1.45) | 0.401 | 0.28 (0.05, 1.7) | 0.163 |
| Only OWT/OB | 2.8 (0.75, 5.26) | 0.123 | 2.69 (1.43, 5.05) | **0.002** | 4.63 (1.73, 7.52) | **0.002** | 2.49 (1.29, 4.79) | **0.007** | 3.92 (1.37, 11.24) | **0.012** | 2.74 (1.64, 4.59) | **0.0002** | 5.75 (1.89, 17.5) | **0.002** |
| Mild Coexisting burden | 1.54 (0.46, 4.27) | 0.478 | 1.97 (1.04, 3.74) | **0.037** | 4.03 (1.06, 7) | **0.008** | 1.7 (0.95, 3.06) | 0.076 | 2.88 (0.93, 8.92) | 0.066 | 2.14 (1.3, 3.51) | **0.003** | 4.09 (1.26, 13.31) | **0.020** |
| Moderate Coexisting burden | 1.83 (0.52, 4.38) | 0.346 | 1.56 (0.83, 2.93) | 0.168 | 4.56 (1.38, 7.74) | **0.005** | 1.25 (0.67, 2.34) | 0.471 | 3.12 (1.09, 8.95) | **0.034** | 2.46 (1.39, 4.36) | **0.002** | 5.63 (1.74, 18.23) | **0.004** |
| Severe Coexisting burden | 1.26 (0.24, 4.04) | 0.788 | 1.92 (0.88, 4.22) | 0.102 | 3.43 (-0.61, 7.48) | 0.096 | 1.49 (0.73, 3.06) | 0.270 | 3.33 (0.8, 13.75) | 0.096 | 1.27 (0.67, 2.4) | 0.467 | 3.29 (0.75, 14.46) | 0.114 |
| **Model 3b:** Adjusted for model 2 variables and household variables ¶ | | | | | | | | | | | | | | |
| Neither | Referent |  | Referent |  | Referent |  | Referent |  | Referent |  | Referent |  | Referent |  |
| Only Mild FI | 1.28 (0.31, 5.3) | 0.732 | 0.72 (0.36, 1.46) | 0.361 | -0.58 (-3.34, 2.18) | 0.677 | 0.82 (0.43, 1.56) | 0.535 | 0.89 (0.24, 3.32) | 0.865 | 1.1 (0.73, 1.65) | 0.643 | 1 (0.26, 3.8) | 0.998 |
| Only Moderate FI | 1.24 (0.29, 5.37) | 0.773 | 1.08 (0.61, 1.93) | 0.794 | 1.65 (-2.13, 5.42) | 0.389 | 0.97 (0.51, 1.83) | 0.919 | 1.81 (0.6, 5.46) | 0.286 | 1.38 (0.86, 2.27) | 0.205 | 2.27 (0.74, 6.97) | 0.151 |
| Only Severe FI | 0.8 (0.14, 4.54) | 0.800 | 0.34 (0.14, 0.82) | **0.017** | -1.09 (-4.68, 2.5) | 0.548 | 0.45 (0.19, 1.06) | 0.066 | 0.27 (0.05, 1.63) | 0.152 | 0.77 (0.41, 1.44) | 0.404 | 0.28 (0.04, 1.72) | 0.165 |
| Only OWT/OB | 2.8 (0.77, 10.26) | 0.119 | 2.81 (1.5, 5.26) | **0.002** | 4.79 (1.98, 7.6) | **0.001** | 2.59 (1.34, 5.02) | **0.005** | 3.98 (1.38, 11.49) | **0.011** | 2.77 (1.66, 4.61) | **0.000** | 5.8 (1.89, 17.77) | **0.002** |
| Mild Coexisting burden | 1.53 (0.47, 4.94) | 0.475 | 2.04 (1.09, 3.82) | **0.027** | 4.13 (1.23, 7.02) | **0.006** | 1.75 (0.98, 3.14) | 0.060 | 2.89 (0.93, 9.02) | 0.067 | 2.17 (1.32, 3.56) | **0.003** | 4.1 (1.25, 13.49) | **0.021** |
| Moderate Coexisting burden | 1.84 (0.53, 6.35) | 0.331 | 1.58 (0.85, 2.96) | 0.149 | 4.68 (1.51, 7.86) | **0.004** | 1.27 (0.69, 2.36) | 0.440 | 1.08 (9.11, 9.94) | **0.036** | 2.5 (1.41, 13.49) | **0.002** | 5.71 (1.73, 18.8) | **0.005** |
| Severe Coexisting burden | 1.26 (0.24, 6.52) | 0.784 | 1.98 (0.89, 4.41) | 0.096 | 3.63 (-0.49, 7.75) | 0.084 | 1.53 (0.73, 3.19) | 0.255 | 3.36 (0.79, 14.32) | 0.100 | 1.31 (0.68, 18.8) | 0.417 | 3.38 (0.74, 15.5) | 0.116 |

All regression analysis accounted for survey weights**. P-value** ≤0.05 is considered statistically significant.

*Abbreviations: OR=odds ratio. CI=confidence interval. FI=food insecurity. OWT/OB=overweight/obesity. SBP=systolic blood pressure. DBP=diastolic blood pressure. AHA/ACC= American Heart Association/American College of Cardiology. JNC=Joint National Committee. ESC=European Society of Cardiology.

†Outcome 1, 3, and 4 (hypertension) were modeled with multinomial logistic regression. Outcome 2 (SBP) was modeled using linear regression.

Hypertension status was defined as: 1) AHA/ACC 2017 guidelines (pre-hypertension 120-129 and DBP <80, hypertension SBP≥130 or DBP≥80); 3)JNC-8 guidelines (pre-hypertension 120-139 and DBP 80-89, hypertension SBP≥140 or DBP≥90); and 4)ESC-2024 guidelines (pre-hypertension 120-139 and DBP 70-89, hypertension SBP SBP≥140 or DPB≥90).

‡ Individual-level covariates: age group (15-19y, 20-29y, 30-39y, 40-49y), physical activity (tertiles of min/week), lactating status (yes/no).

§Household-level covariates: socio-economic position (tertiles), number of children in the household (0, 1-2, ≥3), number of adults in the household (1-2, 3-4, ≥5), area (urban vs rural).

¶Includes same covariates as Model 2 + socio-economic position (tertiles), number of children birthed (0, 1-2, ≥3), number of adults in the household (1-2, 3-4, ≥5), area (urban vs rural).


# **Supplement Table 6:** Characteristics of the sample, stratified by hypertension status^a^

|  |  | **HYPERTENSION STATUS** | | |
| --- | --- | --- | --- | --- |
|  | **Total (N=1538)** | **Normal (N=946, 61%)** | **Pre-hypertension (N=100, 7%)** | **Hypertension (N=492, 32%)** |
| **SBP (mean ± SD) *** | 114.1 ± 13.6 | 108.8 ± 7.6 | 123.3 ± 2.5 | 126.2 ± 14.2 |
| **Food insecurity** |  |  |  |  |
| *None* | 397 (26%) | 239 (25%) | 26 (26%) | 132 (27%) |
| *Mild* | 612 (40%) | 372 (39%) | 39 (39%) | 201 (41%) |
| *Marginal* | 364 (23%) | 226 (24%) | 27 (27%) | 111 (22%) |
| *Severe* | 165 (11%) | 109 (12%) | 8 (8%) | 48 (10%) |
| **BMI (mean ± SD)** | 27.0 ± 5.5 | 25.8 ± 5.1 | 27.7 ± 5.3 | 29.3 ± 5.6 |
| **BMI classification** |  |  |  |  |
| *Underweight* | 36 (2%) | 31 (3%) | 0 (0%) | 5 (1%) |
| *Normal* | 575 (38%) | 434 (46%) | 35 (35%) | 106 (21%) |
| *Overweight* | 523 (34%) | 312 (33%) | 35 (35%) | 176 (36%) |
| *Obese* | 404 (26%) | 169 (18%) | 30 (30%) | 205 (42%) |
| **Coexistence burden (FI + OWT/OB)** |  |  |  |  |
| *Neither* | 144 (9%) | 107 (11%) | 7 (7%) | 30 (6%) |
| *Only FI* | 467 (30%) | 358 (38%) | 28 (28%) | 81 (17%) |
| *Only OWT/OB* | 253 (16%) | 132 (14%) | 19 (19%) | 102 (21%) |
| *Coexistence burden (both)* | 674 (44%) | 349 (37%) | 46 (46%) | 279 (57%) |
| **Age (mean ± SD)** | 30.2 ± 9.3 | 28.0 ± 8.6 | 31.1 ± 9.4 | 34.2 ± 9.3 |
| **Age group** |  |  |  |  |
| *15-19 years* | 240 (16%) | 183 (19%) | 15 (15%) | 42 (9%) |
| *20-29 years* | 511 (33%) | 384 (41%) | 23 (23%) | 104 (21%) |
| *30-39 years* | 480 (31%) | 260 (27%) | 42 (42%) | 178 (36%) |
| *40-49 years* | 307 (20%) | 119 (13%) | 20 (20%) | 168 (34%) |
| **Physical activity (tertiles)**^b^ |  |  |  |  |
| *Low* | 562 (37%) | 327 (35%) | 40 (40%) | 195 (39%) |
| *Middle* | 497 (32%) | 319 (34%) | 32 (32%) | 146 (30%) |
| *High* | 479 (31%) | 300 (31%) | 28 (28%) | 151 (31%) |
| **Lactating status** |  |  |  |  |
| *No* | 1292 (84%) | 764 (81%) | 82 (82%) | 446 (91%) |
| *Yes* | 246 (16%) | 182 (19%) | 18 (18%) | 46 (9%) |
| **Socioeconomic position (tertiles)**^c^ |  |  |  |  |
| *Lower* | 533 (35%) | 333 (35%) | 36 (36%) | 164 (33%) |
| *Middle* | 526 (34%) | 318 (34%) | 34 (34%) | 174 (36%) |
| *Higher* | 479 (31%) | 295 (31%) | 30 (30%) | 154 (31%) |
| **N children in household** |  |  |  |  |
| *0 children* | 171 (11%) | 91 (10%) | 21 (21%) | 117 (34%) |
| *1-2 children* | 790 (51%) | 487 (51%) | 53 (53%) | 276 (56%) |
| *≥3 children* | 577 (38%) | 368 (39%) | 26 (26%) | 99 (20%) |
| **N adults in household** |  |  |  |  |
| *1-2 adults* | 841 (55%) | 520 (55%) | 57 (57%) | 264 (54%) |
| *3-4 adults* | 479 (31%) | 281 (30%) | 31 (31%) | 167 (34%) |
| *≥5 adults* | 218 (14%) | 145 (15%) | 12 (12%) | 61 (12%) |
| **Urban / rural** |  |  |  |  |
| *Urban* | 583 (38%) | 352 (37%) | 30 (30%) | 201 (41%) |
| *Rural* | 955 (62%) | 594 (63%) | 70 (70%) | 291 (59%) |

*Abbreviations: . FI=food security; OWT/OB=overweight/obese; SBP=systolic blood pressure; SD=standard deviation.

^a^N=1538. Results presented in N (%), except for SBP and age presented as mean ± SD.

^b^Physical activity includes minutes/week of moderate and vigorous (multiplied by 2 to convert to moderate) sports activity, as well as bicycle activity. Total minutes were summed and divided into tertiles as low (0-60), middle (61-210) and high (211-4200).

^c^SEP was derived from 9 variables that proxied household and individual assets/socio-economic position. See Supplement Table 3.
